# The Role of Glyceraldehyde-3-Phosphate Dehydrogenase in 2-Ketogluconic Acid Industrial Production Strain *Pseudomonas plecoglossicida* JUIM01

**DOI:** 10.3390/foods14223830

**Published:** 2025-11-08

**Authors:** Lei Sun, Dao-Jiao Tang, Qian-Nan Zhang, Lu-Lu Li, Lei Zhang, Xin-Yi Zan, Feng-Jie Cui, Ling Sun, Wen-Jing Sun

**Affiliations:** School of Food and Biological Engineering, Jiangsu University, Zhenjiang 212013, China; sunlei@ujs.edu.cn (L.S.); 13655603936@163.com (D.-J.T.); zhangqiannan9912@163.com (Q.-N.Z.); 15380664161@163.com (L.-L.L.); 2222418102@stmail.ujs.edu.cn (L.Z.); zxy19880920@163.com (X.-Y.Z.); fengjiecui@163.com (F.-J.C.)

**Keywords:** 2-ketogluconic acid (2KGA), *Pseudomonas plecoglossicida*, glyceraldehyde-3-phosphate dehydrogenase (GAPDH/Gap), *gapA* gene, industrial production strain

## Abstract

The full-length *gapA* gene (1002 bp) was cloned from *Pseudomonas plecoglossicida* JUIM01, an industrial strain used for 2-ketogluconic acid (2KGA) production. The protein encoded by *gapA* (designated Gap) was predicted to be a canonical NAD(P)-dependent glyceraldehyde-3-phosphate dehydrogenase that catalyzes the interconversion between glyceraldehyde-3-phosphate and 1,3-bisphosphoglycerate. Bioinformatics analyses and electrophoretic mobility shift assays suggested that *gapA* is regulated by the transcription factor HexR. Through the knockout and complementation of the gene, along with shake-flask experiments and fermentation in bioreactors, this study demonstrated that the deletion of *gapA* increased the 2KGA production, sugar–acid conversion rate, molar yield, and productivity of *P. plecoglossicida* JUIM01 by 5.7–6.6% without affecting cell growth, highlighting the mutant’s significant industrial potential.

## 1. Introduction

The cytoplasmic membrane of *Pseudomonas* species harbors a direct glucose oxidation system, mainly consisting of a pyrroloquinoline quinone (PQQ)-dependent glucose dehydrogenase (Gcd) and a flavin adenine dinucleotide (FAD)-dependent gluconate dehydrogenase (Gad). Gcd oxidizes glucose to gluconic acid, and Gad further oxidizes gluconic acid to 2-ketogluconic acid (2KGA) in the periplasmic space [1,2,3,4]. Additionally, *Pseudomonas* strains generally exhibit strong adaptability to diverse physicochemical and nutritional environments, as well as significantly tolerance to endogenous and exogenous stresses [5,6,7,8,9,10]. Hence, they are widely used in the industrial fermentation production of 2KGA. 2KGA has a broad range of industrial applications. Among these applications, the most important one is as a precursor for the synthesis of the food antioxidant d-isoascorbic acid (d-erythorbate) and its salts [11,12,13,14,15].

**Figure 1:**
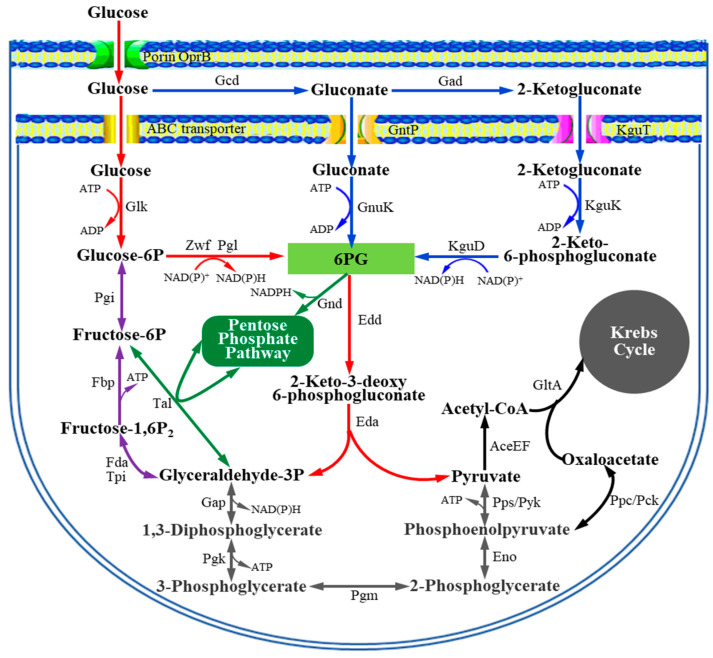
Glucose metabolism in *Pseudomonas* deduced from gene annotations and functional analysis. 6PG, 6-phosphogluconate.

Based on the cellular localization of biochemical reactions, glucose metabolism in *Pseudomonas* can be divided into two components, namely the extracellular oxidation pathway and the intracellular degradation pathway. Since the oxidation of glucose occurs in the periplasmic space and the final product of oxidation is 2KGA, this metabolic pathway can be referred to as the glucose extracellular oxidation pathway or the 2KGA synthesis pathway of *Pseudomonas*. Corresponding to the extracellular oxidation pathway, the intracellular degradation pathway involves a series of biochemical reactions for the phosphorylation of glucose, gluconic acid, and 2KGA (imported from the periplasmic space) and the catabolism of their common phosphorylated product 6-phosphogluconate (6PG) in the cytoplasm [16,17,18,19]. There is no 6-phosphofructokinase in *Pseudomonas* bacteria; hence, they cannot catabolize glucose through the Embden–Meyerhof–Parnas (EMP) pathway in the cytoplasm. Instead, they mainly utilize glucose through a metabolic structure known as the ED–EMP cycle [20,21,22,23], which integrates a series of metabolic reactions from the Entner–Doudoroff (ED) pathway, an incomplete EMP pathway, and the pentose-phosphate (PP) pathway, with glyceraldehyde 3-phosphate as the key metabolic product linking the three pathways in this cycle (Figure 1). Furthermore, glyceraldehyde 3-phosphate is one of the key metabolic products that link the ED–EMP cycle to the Krebs cycle (TCA cycle).

Glyceraldehyde-3-phosphate dehydrogenase (GAPDH/Gap) is a housekeeping enzyme of energy metabolism conserved in virtually all organisms. Type I glyceraldehyde-3-phosphate dehydrogenase (GAPDH-I) catalyzes the interconversion of glyceraldehyde-3-phosphate and 1,3-bisphosphoglycerate, which is a central step in glycolysis and gluconeogenesis [24]. It utilizes NAD (EC 1.2.1.12), NADP (EC 1.2.1.13) or either cofactor (EC 1.2.1.59) [25,26,27]. GAPDH-I, located in the cytoplasm, typically exists as a homotetrameric configuration, with a molecular mass of about 37 kDa per subunit [28]. The protein contains an N-terminal NAD(P)-binding domain and a C-terminal catalytic domain. The primarily N-terminal NAD(P)-binding domain contains a Rossmann fold which combines with the catalytic cysteine-containing C-terminus to form a catalytic cleft [29].

Under specific fermentation conditions, the extracellular oxidation and intracellular degradation pathways of glucose metabolism in *Pseudomonas* maintain a dynamic balance, resulting in a relatively stable 2KGA yield (glucose to 2KGA conversion rate) [3,12,30]. *Pseudomonas plecoglossicida* JUIM01 is currently the strain used in China for the industrial production of 2KGA [3,12,30], and it has been proven to be a biosafe and efficient producer. However, its stress resistance and production efficiency under adverse environmental conditions (e.g., high temperature, high acidity, and high sugar concentration) still require further improvement [31]. Multiple genetic manipulation strategies have been attempted. However, most successful attempts to increase 2KGA production in *P. plecoglossicida* JUIM01 have also adversely affected its growth [30,32,33,34]. Therefore, further enhancing 2KGA production without compromising strain growth remains a challenge. Given the important role of glyceraldehyde-3-phosphate dehydrogenase in the intracellular degradation pathway of glucose in *Pseudomonas*, this study aimed to develop a better understanding of the structure, function, and expression regulation of the enzyme Gap in *P. plecoglossicida* JUIM01. It also aimed to elucidate the role of the *gapA* gene encoding Gap in maintaining the balance between the extracellular oxidation and intracellular degradation of glucose. This is expected to provide theoretical support for improving the stress resistance and production efficiency of 2KGA production strains.

## 2. Materials and Methods

### 2.1. Strains, Plasmids, Media and Cultivation

The bacterial strains and plasmids used in this study are listed in Table 1.

Media used in this study are listed in Table 2. *Escherichia coli* strains were cultured in LB medium or on LB agar plates at 37 °C. Antibiotics (25 µg/mL kanamycin sulfate or 50 µg/mL ampicillin) were supplemented when required. *P. plecoglossicida* strains were initially activated on agar plates, then inoculated into 50 mL of seed medium and cultured at 32 °C and 265 rpm for 24 h, when both glucose (substrate) and 2KGA (product) in the seed medium were depleted, as they were utilized by the strains as carbon sources for growth. The fermentation of 2KGA in 500 mL flasks was conducted by inoculating 10% (*v*/*v*) of the seed culture into 40 mL of fermentation medium, and fermenting at 32 °C and 265 rpm until the substrate glucose was depleted and 2KGA production reached its maximum. The 2KGA fermentation in 10 L bioreactors (Green Bio-engineering Co., Ltd., Zhenjiang, China) was conducted by inoculating 0.6 L of the seed culture into 6 L of fermentation medium, and fermenting at 32 °C and 400 rpm, with an aeration rate of 7.2 L/min, until the substrate glucose was depleted and 2KGA production reached its maximum.

### 2.2. Bioinformatics Analyses of the gapA Gene and Gap Protein in P. plecoglossicida JUIM01

The genomic DNA of *P. plecoglossicida* JUIM01 was extracted using a Bacterial Genomic DNA Kit (Beyotime, Shanghai, China). The target sequence, encompassing the *gapA* gene and *edd* operon, was amplified with PCR using the primers *gapA/edd*-F and *gapA/edd*-R (Table S1) with the genome as the template. The PCR product was sequenced by Sangon Biotech Co. (Shanghai, China). ORF Finder (https://www.ncbi.nlm.nih.gov/orffinder/, accessed on 25 September 2025) was used to predict the open reading frame of *gapA*. NCBI tools (https://blast.ncbi.nlm.nih.gov/Blast.cgi, accessed on 25 September 2025) were used to analyze the identity of *gapA* and the encoded amino acid sequence. The conserved domain of Gap was predicted using the Conserved Domain Search Service tool (CD Search) (https://www.ncbi.nlm.nih.gov/Structure/cdd/wrpsb.cgi, accessed on 25 September 2025). The AlphaFold Protein Structure Database (https://alphafold.ebi.ac.uk/, accessed on 25 September 2025) was used to predict the tertiary structure of the Gap protein. BPROM (http://www.softberry.com/berry.phtml?topic=bprom&group=programs&subgroup=gfindb, accessed on 25 September 2025) and BDGP (http://www.fruitfly.org/seq_tools/promoter.html, accessed on 25 September 2025) were used to analyze the promoter region. Molecular docking simulations of the Gap protein with NAD, glyceraldehyde-3-phosphate, and 1,3-bisphosphoglycerate were performed using AutoDock Vina v1.2.x (Scripps Research, La Jolla, CA, USA).

### 2.3. Construction of the gapA-Knockout Mutant and Its Gene Complementation Strain Derived from P. plecoglossicida JUIM01

A *gapA*-knockout mutant of *P. plecoglossicida* JUIM01 (named JUIM01Δ*gapA*) was constructed using the plasmid pK18*mobsacB* via a two-step homologous recombination method [32]. Two homologous arms flanking the *gapA* gene were amplified from JUIM01 genomic DNA using the primer pairs *gapA*-P1/*gapA*-P2 and *gapA*-P3/*gapA*-P4 (Table S1). They were then fused by overlap extension PCR using the primers *gapA*-P1/*gapA*-P4, and ligated into linearized pK18*mobsacB* to construct the recombinant suicide plasmid pK18*mobsacB*-Δ*gapA* (Table 1). The plasmid was electroporated (1.2 kV for 5 mS) into freshly prepared JUIM01 competent cells. The mutant strain was selected through two rounds of homologous recombination: first on LB plates containing 25 μg/mL kanamycin, followed by counter-selection on LB agar plates with 10% sucrose. The positive clones were confirmed through colony PCR (Figure S2) and sequencing. Afterwards, *gapA* was amplified and integrated between the *Hin*d III and *Bam*H I restriction sites of the expression vector pBBR1MCS-2 using the digestion–ligation method to construct the recombinant plasmid pBBR1MCS-2-*gapA* for gene complementation (Table 1 and Figure S3). The corresponding *gapA* complementation strain (named JUIM01Δ*gapA-gapA*) was constructed by transforming the recombinant plasmid pBBR1MCS-2-*gapA* into JUIM01Δ*gapA*.

### 2.4. 5′-Rapid Amplification of cDNA Ends (5′-RACE)

The transcription start site (TSS) of *gapA* was determined using a 5′-RACE kit (Sangon Biotech, Shanghai, China) with the primers shown in Table S1. The cDNA of *P. plecoglossicida* JUIM01 was synthesized with reverse transcription using its total RNA as the template, and *gapA*-RT1/*gapA*-RT2 as the primers. The resulting cDNA was then digested with RNase H. A poly-C tail was added using deoxynucleotidyl transferase. The second-run PCR was conducted using the above PCR product as the template and NR1/Adaptor and NR2/Outer as the primers (Table S1). The PCR product with a poly-A tail added was ligated with pMD20-T to construct the plasmid pMD20-T-*gapA*. pMD20-T-*gapA* was transferred to *E. coli* JM109 to screen the positive transformants with LB plates containing 50 μg/mL ampicillin for further sequencing.

### 2.5. Electrophoretic Mobility Shift Assay (EMSA)

Electrophoretic mobility shift assays (EMSA) were conducted as previously reported [37], with some modifications. The *hexR* gene from *P. plecoglossicida* JUIM01 was heterologously expressed in *E. coli* (Table 1). The recombinant protein was purified using Ni-NTA affinity chromatography. The primers G1 and G3 (Table S1) were used to amplify the DNA probe P*_gapA_* labeled with biotin at the 5′ end, and G2 and G3 (Table S1) were used to amplify the unlabeled DNA probe P*_gapA_*. The purified protein and the probes were used to identify specific binding between HexR and the *gapA* promoter region. The reaction systems of the EMSA are shown in Table S2.

### 2.6. Analytical Methods

The growth of *P. plecoglossicida* strains was assessed by measuring the optical density at 650 nm (OD_650nm_) using a Biospec-1601 spectrophotometer (Shimadzu, Kyoto, Japan). An OD_650nm_ of 1.0 represents 0.575 g of dry cell weight per liter [30]. The glucose concentration was measured using an SBA-40C biosensor (Biology Institute of Shandong Academy of Sciences, Jinan, China) at room temperature. The concentration of 2KGA was determined using the iodometric method developed by our group [38].

### 2.7. Statistical Analysis

All the experiments included three biological replicates. The data are presented as the mean ± standard deviation (*n* = 3) and were analyzed using a one-way analysis of variance (ANOVA). Statistical significance was determined based on the *p* value (*α* = 0.05).

## 3. Results and Discussion

### 3.1. Identification of the Gene Encoding Glyceraldehyde-3-Phosphate Dehydrogenase in P. plecoglossicida JUIM01

Based on the results of genome sequencing and annotation, the *gapA* gene encoding glyceraldehyde-3-phosphate dehydrogenase in *P. plecoglossicida* JUIM01 was cloned using the primers *gapA/edd*-F and *gapA/edd*-R (Table S1). The full-length sequence is 1002 bp, with a start codon ATG and a stop codon TGA. Gene sequence alignment revealed that the JUIM01 *gapA* gene shared 91.27%, 91.27%, 91.37%, 91.37%, and 95.11% sequence identity with orthologs from *P. guariconensis* (CP162012.1), *P. putida* (AP022227.1), *Pseudomonas* sp. BYT-1 (CP072559.1), *Pseudomonas* sp. BYT-5 (CP097489.1), and *Pseudomonas* sp. p1 (2021b) (CP083746.1), respectively. The results showed that the amino acid sequence identity of the protein encoded by *gapA* in JUIM01 with the corresponding GAPDH-Is of *P. guariconensis* (WP_196144501.1), *P. guariconensis* (WP_196155289.1), *Pseudomonas* sp. p1 (2021b) (WP_224456780.1), *P. putida* (EKT4467476.1), and *Pseudomonas* (WP_084856669.1) was 97.60%, 97.90%, 97.90%, 100%, and 100%, respectively. Therefore, the JUIM01 Gap protein was predicted to be a cytoplasm, hydrophilic, acidic GAPDH-I consisting of 333 amino acids, with a theoretical molecular mass of 36.15 kDa. Conservative domain analysis further predicted that the protein encoded by JUIM01 *gapA* is a GAPDH-I that catalyzes the NAD(P)-dependent oxidative phosphorylation of glyceraldehyde-3-phosphate to 1,3-diphosphoglycerate. Its N-terminal domain is a Rossmann NAD(P)-binding fold, and the C-terminal domain is a mixed alpha/antiparallel beta fold (Figure S1). GAPDHs usually contains two major domains, the NAD+ binding domain (amino acids 1–150) and the catalytic or glyceraldehyde-3-phosphate domain (amino acids 151–335) [28]. The molecular docking predictions for the Gap of *P. plecoglossicida* JUIM01 with NAD, glyceraldehyde-3-phosphate, or 1,3-bisphosphoglycerate also aligned with these findings (Figure S2).

### 3.2. Analysis of the Promoter Region of gapA in P. plecoglossicida JUIM01

**Figure 2:**
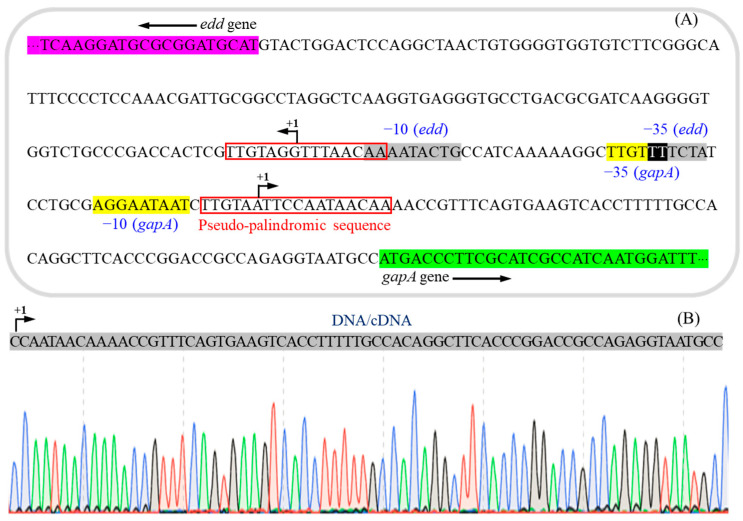
The prediction of the promoter region of *gapA* (**A**) and the determined transcription start site of *gapA* by 5′-RACE (**B**).

The prediction for the promoter region of *gapA* in *P. plecoglossicida* JUIM01 is summarized in Figure 2A. In JUIM01, *gapA* is adjacent to the *edd* gene, which encodes 6-phosphogluconate dehydratase, but they are transcribed in opposite directions. Within the *edd-gapA* intergenic region, the sequence of the −10 box (RNA polymerase binding site) in the promoter of *edd* is CAGTATTTT (its reverse complement is AAAATACTG), and the sequence of the −35 box (RNA polymerase σ factor recognition site) is TAGAAA (its reverse complement is TTTCTA). The transcription start site (+1) of *edd* is located 129 bp upstream of the gene and lies within a pseudo-palindromic consensus sequence (TTGT-N_7_-ACAA) [30]. Using the online analysis software BPROM, the predicted −10 box sequence of the promoter of *gapA* is AGGAATAAT, and the −35 box sequence is TTGTTT, which overlaps with the *edd* gene’s −35 box by 2 bp. Its predicted transcription start site (+1) is located 75 bp upstream of *gapA* and lies within a pseudo-palindromic consensus sequence (TTGT-N_10_-ACAA) [30]. The transcription start site (+1) determined by 5′-RACE is located 72 bp upstream of *gapA*, showing a 3 bp deviation from the predicted site, but it is also located within the pseudo-palindromic consensus sequence (TTGT-N_10_-ACAA) (Figure 2B).

HexR is a ubiquitous global central carbon metabolism regulator in *Pseudomonas* that regulates the expression of genes related to glucose uptake, glucose phosphorylation, and glucose catabolism via the ED pathway [17,39,40,41,42]. The pseudo-palindromic consensus sequence (5′-TTGT-N_7/8_-ACAA-3′) is its binding site within the promoter regions of target genes (such as the *edd* operon, *zwf/pgl/eda* operon, and *gap-1*) in *P. putida* and *P. fluorescens* [17,41]. Our previous findings demonstrated that the *edd* gene is also transcriptionally regulated by HexR in *P. plecoglossicida* [30]. The analysis of the *gapA* promoter region described above suggests that *gapA* in *P. plecoglossicida* is likely under the same regulatory control by HexR.

**Figure 3:**
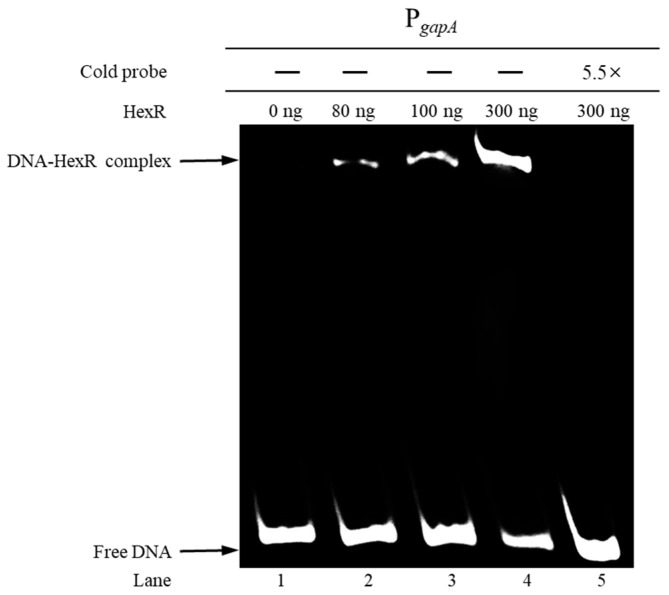
EMSA analysis of the specific binding between HexR and the *gapA* promoter region in *P. plecoglossicida*.

The EMSA confirmed the above speculation. As shown in Figure 3, the 5′-biotin-labeled DNA probe P*_gapA_* (which contains the *gapA* promoter region sequence) could bind to varying concentrations (80–300 ng) of the HexR protein, resulting in mobility shifts (Lanes 2–4). In contrast, when no HexR was added to the reaction system (Lane 1) or when an excess of unlabeled competitive probe was added (Lane 5), only the free DNA band was observed. These results demonstrate that the transcription factor HexR could specifically bind to the *gapA* promoter region in *P. plecoglossicida*.

### 3.3. The Role of gapA in the Growth and Metabolism of P. plecoglossicida JUIM01

**Figure 4:**
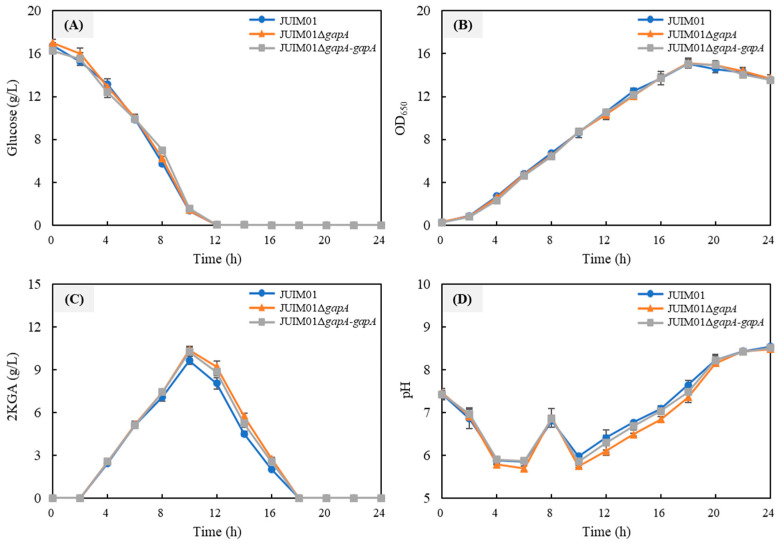
Time course of the cultivation of JUIM01, JUIM01Δ*gapA* and JUIM01Δ*gapA-gapA* in seed medium in shake flasks. (**A**) Glucose consumption; (**B**) cell growth; (**C**) 2-ketogluconic acid (2KGA) metabolism; and (**D**) pH of the culture broth.

To elucidate the role of *gapA* in the growth and metabolism of *P. plecoglossicida*, a *gapA* gene deletion mutant (JUIM01Δ*gapA*) and its complementation strain (JUIM01Δ*gapA-gapA*) were constructed. The PCR validations are shown in Figures S3 and S4. The differences in growth and metabolism among the parental strain JUIM01, the *gapA* deletion mutant, and the complemented strain were then compared in seed medium containing 18 g/L glucose as the carbon source (Figure 4). As shown in Figure 4A, JUIM01, JUIM01Δ*gapA*, and JUIM01Δ*gapA-gapA* were all capable of rapidly utilizing glucose, depleting it within 12 h, with a glucose consumption rate exceeding 1.5 g/L/h. The cell growth of the three strains was nearly identical (Figure 4B). Regarding 2KGA metabolism, the accumulation of 2KGA in all three strains peaked at 10 h, after which the strains reutilized 2KGA as the alternative carbon source until it was depleted [3]. Interestingly, the knockout of *gapA* increased the maximum 2KGA accumulation in *P. plecoglossicida* JUIM01 from 9.64 g/L to 10.28 g/L, indicating that the absence of *gapA* is more favorable for 2KGA production in *P. plecoglossicida*. This was further confirmed by the 2KGA production profile of JUIM01Δ*gapA-gapA*, where *gapA* complementation restored 2KGA production to near-parental levels (Figure 4C). Correspondingly, the pH of JUIM01Δ*gapA* was the lowest among the three strains due to it having the highest 2KGA (an organic acid) production (Figure 4D).

### 3.4. 2KGA Production Performance of P. plecoglossicida JUIM01ΔgapA

**Figure 5:**
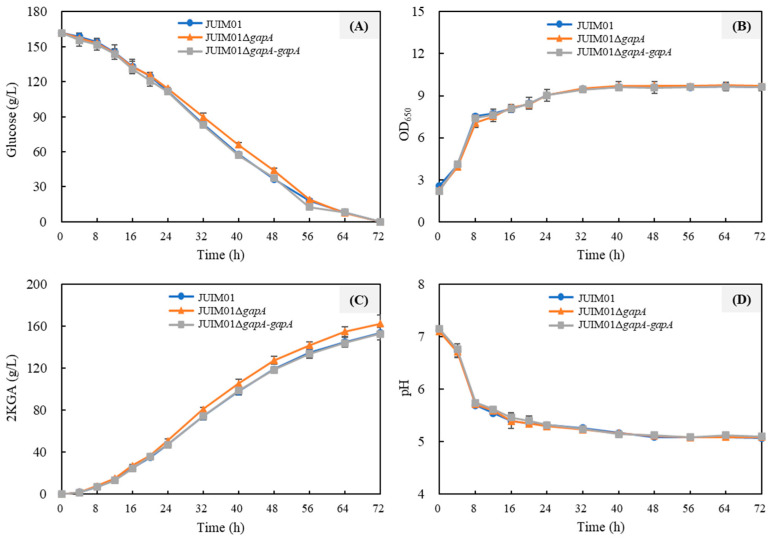
Time course of 2KGA fermentation under shake-flask culture conditions. (**A**) Glucose consumption; (**B**) cell growth (OD_650nm_); (**C**) 2-ketogluconic acid (2KGA) production; and (**D**) pH of the fermentation broth.

**Figure 6:**
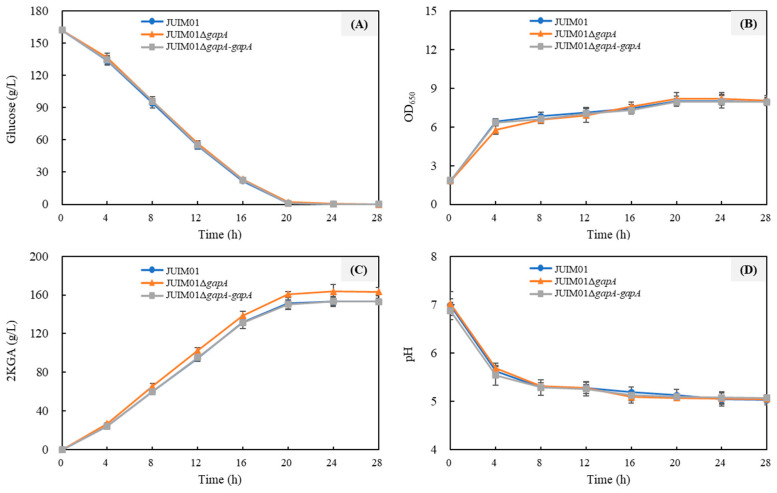
Time course of 2KGA fermentation in 10 L bioreactors. (**A**) Glucose consumption; (**B**) cell growth (OD_650nm_); (**C**) 2-ketogluconic acid (2KGA) production; and (**D**) pH of the fermentation broth.

To further confirm that the deletion of *gapA* is beneficial for 2KGA production and to clarify its impact on 2KGA fermentation by *P. plecoglossicida*, we compared the performance of JUIM01, JUIM01Δ*gapA*, and JUIM01Δ*gapA-gapA* in shake flasks (Table 3 and Figure 5) and in 10 L bioreactors (Figure 6). When using 162 g/L glucose as the substrate, the glucose was nearly depleted in the fermentation broth of all three strains after 72 h of shake-flask fermentation, and the OD_650nm_ values reached their peak and remained stable. JUIM01 and JUIM01Δ*gapA-gapA* showed similar fermentation performance, whereas JUIM01Δ*gapA* exhibited the highest 2KGA titer (163.43 g/L), sugar–acid (glucose–2KGA) conversion rate (100.27 g/100 g glucose), molar yield (93.03%), and productivity (2.26 g/L/h). These values were significantly higher (*p* < 0.05) by more than 5.7% compared to the other two strains.

The superiority of *P. plecoglossicida* JUIM01Δ*gapA* for 2KGA production was more evident in the 10 L bioreactors. 2KGA is synthesized from glucose through two oxidation reactions. Therefore, sufficient aeration is crucial for 2KGA fermentation [4,43]. The maximum 2KGA titers of the three strains in bioreactors were similar to those in shake-flask fermentation, but their productivities were significantly enhanced with better aeration, and the fermentation time was reduced to within 28 h. Specifically, after 24 h of fermentation, the 2KGA production of JUIM01Δ*gapA* reached its peak value of 163.42 g/L, with a productivity of 6.81 g/L/h, representing a 6.6% increase (*p* < 0.05) compared to the other two strains (Figure 6). The yield of JUIM01Δ*gapA* reached 93.58% (93.58 mol 2KGA/100 mol glucose), which is among the highest levels reported in the past 15 years [44].

So far, we have identified that the knockout of several genes can enhance the accumulation of 2KGA in *Pseudomonas* (Table 4), such as *kguT* (which encodes 2-ketogluconate transporter) [32,33], *kguD* (which encodes 2-keto-6-phosphogluconate reductase) [33], *kguK* (which encodes 2-ketogluconate kinase) [33,34], *kguE* (which encodes a putative epimerase) [33], and *edd* (which encodes 6-phosphogluconate dehydratase) [30]. Among these, the knockout of *kguT* or *kguD* promotes 2KGA accumulation, but results in a reduced maximum cell density of the production strain, as the absence of *kguT* or *kguD* prevents *Pseudomonas* from utilizing 2KGA as a carbon source for growth. The knockout of *kguK* or *kguE* does not reduce the maximum cell density, but slows the growth rate of the production strain due to the delayed utilization of 2KGA caused by the loss of *kguK* or *kguE*. The knockout of *edd* significantly inhibits the growth of *Pseudomonas*, as it blocks glucose metabolism via the ED pathway and disables the strain’s ability to utilize 2KGA. A notable distinction of *gapA* from the aforementioned genes is that its knockout enhanced the 2KGA production in *Pseudomonas* without adversely affecting cell growth. This characteristic makes JUIM01Δ*gapA* a promising candidate for industrial 2KGA production. However, the underlying mechanism by which *gapA* deletion enhances 2KGA production remains unclear. Beyond its initially characterized role in glycolysis, GAPDH is recognized as a multifunctional enzyme (although primarily documented in higher organisms) [28,45,46,47]. Furthermore, genomic sequencing data indicate that *P. plecoglossicida* JUIM01 may harbor additional genes encoding GAPDH homologs (unpublished data). These factors collectively complicate our interpretation of the mechanisms underlying *gapA*. Perhaps transcriptomic or metabolomic analyses could provide new insights for this question.

## 4. Conclusions

We cloned a full-length 1002 bp gene, *gapA*, from the industrial 2KGA production strain *P. plecoglossicida* JUIM01. The encoded protein Gap was predicted to be a canonical NAD(P)-dependent GAPDH-I that contains the conserved domains of GAPDH-Is and is capable of catalyzing the interconversion between glyceraldehyde-3-phosphate and 1,3-bisphosphoglycerate. Analyses of the promoter region revealed that the transcription start site is located 72 bp upstream of *gapA*, within a pseudo-palindromic sequence (TTGT-N_10_-ACAA). This suggests that, similar to *P. putida* and *P. fluorescens*, *gapA* in *P. plecoglossicida* is likely regulated by the transcription factor HexR. The EMSA confirmed this speculation. Through the knockout and complementation of *gapA*, along with shake-flask experiments and fermentation in bioreactors, this study demonstrated that the deletion of *gapA* increased the 2KGA production, sugar–acid conversion rate, molar yield, and productivity of *P. plecoglossicida* JUIM01 by 5.7–6.6% without affecting cell growth. Given that *P. plecoglossicida* JUIM01 is already a high-yield industrial 2KGA-producing strain (molar yield > 88%), this 5.7–6.6% improvement is substantial, underscoring the significant industrial potential and economic value of the JUIM01Δ*gapA* mutant. The findings of this study also provide guidance for the metabolic engineering of other *Pseudomonas* species.

## Figures and Tables

**Table 1:** Strains and plasmids used in this study.

Strains and Plasmids	Description	Source
**Strains**		
*P*. *plecoglossicida* JUIM01	2-Ketogluconate industrial producing strain	Our lab
*E. coli* JM109	General cloning strain	TaKaRa (Beijing, China)
*E. coli* BL21(DE3)	Heterologous gene expression strain	TaKaRa
*E. coli* JM109/pK18*mobsacB*-Δ*gapA*	JM109 containing vector pK18*mobsacB*-Δ*gapA*	This work
*E. coli* JM109/pBBR1MCS-2-*gapA*	JM109 containing vector pBBR1MCS-2-*gapA*	This work
*E. coli* JM109/pMD20-T-*gapA*	JM109 containing vector pMD20-T-*gapA*	This work
*P*. *plecoglossicida* JUIM01Δ*gapA*	*gapA*-knockout mutant of JUIM01	This work
*P*. *plecoglossicida* JUIM01Δ*gapA-gapA*	*gapA*-complemented strain of JUIM01Δ*gapA*	This work
*E. coli* BL21(DE3)/pET-24b-*hexR*	Heterologous *hexR* gene expression strain	This work
**Plasmids**		
pK18*mobsacB*	Mobilizable *E*. *coli* vector, Kan^r^, Suc^s^	[35]
pBBR1MCS-2	*E. coli*-*Pseudomonas* shuttle vector, Kan^r^	[36]
pMD20-T	T-vector, Amp^r^, *lacZ*	TaKaRa
pET-24b(+)	*E*. *coli* expression vector, Kan^r^	TaKaRa
pK18*mobsacB*-Δ*gapA*	pK18*mobsacB* containing incomplete *gapA* sequence of JUIM01	This work
pBBR1MCS-2-*gapA*	pBBR1MCS-2 containing the *gapA* of JUIM01	This work
pMD20-T-*gapA*	pMD20-T containing the promoter region of *gapA* (for 5′-RACE)	This work
pET-24b-*hexR*	pET-24b(+) containing the *hexR* of JUIM01	This work

**Table 2:** Media used in this study.

Medium	Component (g/L) and pH	Description
LB medium and agar plate	Peptone 10, beef extract 5, NaCl 5 without/with agar 20, pH of 7.0	For *E. coli* culture
Activation plate	Peptone 10, beef extract 5, NaCl 5, and agar 20, pH of 7.0	For activation of *P*. *plecoglossicida*
Seed medium	Glucose 18, corn syrup powder 5, urea 2, KH_2_PO_4_ 2, MgSO_4_·7H_2_O 0.5, and CaCO_3_ 5, pH of 7.0	For analysis of the role of *gapA* in the growth and metabolism of *P*. *plecoglossicida* (Section 3.3), and to for seed culture preparation
Fermentation medium	Glucose 162, corn syrup powder 10, and CaCO_3_ 45, pH of 6.7	For 2KGA fermentation in shake flasks and bioreactors (Section 3.4)

**Table 3:** Comparison of 2KGA production performance among the strains JUIM01, JUIM01Δ*gapA*, and JUIM01Δ*gapA-gapA* under shake-flask culture conditions.

Strains	JUIM01	JUIM01Δ*gapA*	JUIM01Δ*gapA-gapA*
Initial Glucose (g/L)	162.00 ± 0.00	162.00 ± 0.00	162.00 ± 0.00
Residual Glucose (g/L)	0.01 ± 0.00	0.00 ± 0.00	0.03 ± 0.00
Maximum Cell Concentration (OD_650nm_)	9.66 ± 0.31	9.73 ± 0.12	9.64 ± 0.21
2KGA Production (g/L)	153.68 ± 6.48	162.43 ± 8.02 *	152.84 ± 5.94
2KGA Yield (g/100 g)	94.86 ± 0.04	100.27 ± 0.05 *	94.35 ± 0.04
2KGA Yield (mol/100 mol)	88.02 ± 0.04	93.03 ± 0.05 *	87.54 ± 0.03
Fermentation Period (h)	72.0	72.0	72.0
2KGA Productivity (g/L/h)	2.13 ± 0.09	2.26 ± 0.11 *	2.12 ± 0.08

* The asterisk indicates a statistically significant difference compared to the other datasets (*p* < 0.05).

**Table 4:** Genes whose knockout may enhance 2KGA production in *Pseudomonas*.

Gene	Protein	Adverse Impact on Cell Growth	Mechanism	Reference
*kguT*	2-Ketogluconate transporter	Yes	Mutant cannot utilize 2KGA	[32,33]
*kguD*	2-Keto-6-phosphogluconate reductase	Yes	Mutant cannot utilize 2KGA	[33]
*kguK*	2-Ketogluconate kinase	Yes	Slower utilization of 2KGA	[33,34]
*kguE*	A putative epimerase (not clear)	Yes	Slower utilization of 2KGA	[33]
*edd*	6-Phosphogluconate dehydratase	Yes	Mutant cannot utilize 2KGA; ED pathway is blocked	[30]
*gapA*	Glyceraldehyde-3-phosphate dehydrogenase	No	Not clear	This work

## Data Availability

The original contributions presented in this study are included in the article/Supplementary Materials. Further inquiries can be directed to the corresponding authors.

## Supplementary Materials

The following supporting information can be downloaded at: https://www.mdpi.com/article/10.3390/foods14223830/s1, Table S1: Primers used in this study; Table S2: EMSA reaction systems; Figure S1: The predicted tertiary structure of Gap in *Pseudomonas plecoglossicida* JUIM01. Protein: Glyceraldehyde-3-phosphate dehydrogenase. Gene: B7H17_13130. Source Organism: *Pseudomonas putida*. UniProt: A0A1X0ZWI9. Average pLDDT: 97.31 (very high). Sequence length: 333; Figure S2: Molecular docking predictions for the Gap of *Pseudomonas plecoglossicida* JUIM01 with (A) glyceraldehyde-3-phosphate, (B) 1,3-bisphosphoglycerate, (C) NAD, and (D) both glyceraldehyde-3-phosphate (blue) and NAD (purple); Figure S3: PCR validation of *gapA* knockout of *Pseudomonas plecoglossicida* JUIM01. M, DL 5000 DNA Marker; Lane 1, JUIM01; Lane 2, JUIM01Δ*gapA*; Figure S4: Restriction enzyme digestion verification of the recombinant expression plasmid pBBR1MCS-2-*gapA* for *gapA* complementation. The recombinant plasmid pBBR1MCS-2-*gapA* was verified through the digestion of the gene fragment with *Bam*H I and *Hin*d III. M, DL 15,000 DNA Marker; Lane 1, the single enzyme (*Bam*H I) digestion product of the recombinant plasmid; Lane 2, the double enzyme (*Bam*H I/*Hin*d III) digestion products of the recombinant plasmid.

## Abbreviations

The following abbreviations are used in this manuscript:

2KGA	2-Ketogluconic acid
GAPDH-I	Glyceraldehyde-3-phosphate dehydrogenase, type I
EMP	Embden–Meyerhof–Parnas
ED	Entner–Doudoroff
6PG	6-Phosphogluconate
5′-RACE	5′-Rapid amplification of cDNA ends
EMSA	Electrophoretic mobility shift assay
OD_650nm_	Optical density at 650 nm
