# Supplementary material for: The Role of Glyceraldehyde-3-Phosphate Dehydrogenase in 2-Ketogluconic Acid Industrial Production Strain Pseudomonas plecoglossicida JUIM01
Source: Foods. 2025 Nov 8;14(22):3830. doi: 10.3390/foods14223830 (PMC12651266; doi:10.3390/foods14223830)
Supplement: Supplementary file 1 [file foods-14-03830-s001.zip › foods-3924169-supplementary.pdf]

Supplementary materials

The Role of Glyceraldehyde-3-phosphate Dehydrogenase in 2-Ketogluconic Acid Industrial Production Strain *Pseudomonas plecoglossicida* JUIM01

Table S1. Primers used in this study

Primers	Sequences (5′→3′)	Description (restriction sites)
gapA/edd-F	GTGAGGAAGACGGTATCGAAGCGGGACT	To amplify the sequence including *gapA* gene and *edd* operon from *Pseudomonas plecoglossicida* JUIM01
gapA/edd-R	GCGAGCAGGTAACGAAGGGATGCACGAA	
pK18-F	GACAAGGGAAAACGCAAG	For amplification of linearized vector pK18*mobsacB*
pK18-R	GGGATAACGCAGGAAAGAAC	
gapA-P1	CTTTCCTGCGTTATCCCCGTAGAAAGTACAGGTGCCCG	For amplification of the left homologous arm of *gapA*, containing homologous sequence to the pK18*mobsacB* plasmid (indicated by underlining)
gapA-P2	CGCCAGACAGTTATCCAGTGCGGCCAAATCCATTGA	
gapA-P3	TCAATGGATTTGGCCGCACTGGATAACTGTCTGGCG	For amplification of the right homologous arm of *gapA*, containing homologous sequence to the pK18*mobsacB* plasmid (indicated by underlining)
gapA-P4	TGCGTTTTCCCTTGTCAGCCAGTCCATCGCCT	
gapA-P5	CCCAAGCTTATGACCCTTCGCATCG	For amplification of *gapA* with restriction enzyme site (*Hin*d III, underlined)
gapA-P6	CGCGGATCCTCAGCGGGCGTTGCACAAC	For amplification of *gapA* with restriction enzyme site (*Bam*H I, underlined)
gapA-RT1	GAGCACCTGGTCATTGGTATAGG	Primers for 5′-RACE
gapA-RT2	CTTGCTCGATGCCGAACTC	
NR1	CCGTTGACCGTCAGGCTCTC	
NR2	TGGGCGTTCATCGCACTGT	
Adaptor	GCTGTCAACGATACGCTACGTAACGGCATGACAGTGGGIIGGGIIGGGIIG	
Outer	GCTGTCAACGATACGCTACGTAAC	
G1	5'-Biotin-GGCTTGTTTTCTATCCTGCGAG	Primers for EMSA
G2	GGCTTGTTTTCTATCCTGCGAG	
G3	ATTGATGGCGATGCGAAGGGT	

5'-Biotin- indicates that the sequence is labeled with biotin at its 5' end.

Table S2. EMSA reaction systems

Component\Lane	1	2	3	4	5

Nuclease-Free Water (μL)	7	6.2	6	6.61	1.61
5× Binding Buffer (μL)	2	2	2	2	2
HexR (ng)	/	80	100	300	300
Unlabeled Probe (ng)	/	/	/	/	570
5' Biotin-Labeled Probe (ng)	104	104	104	104	104
Total Volume (μL)	10	10	10	10	10

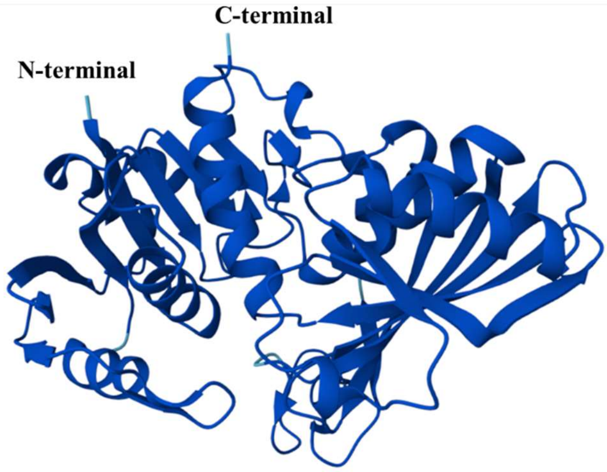

Figure S1. The predicted tertiary structure of Gap in *Pseudomonas plecoglossicida* JUIM01. Protein: Glyceraldehyde-3-phosphate dehydrogenase. Gene: B7H17_13130. Source Organism: *Pseudomonas putida*. UniProt: A0A1X0ZWI9. Average pLDDT: 97.31 (very high). Sequence length: 333.

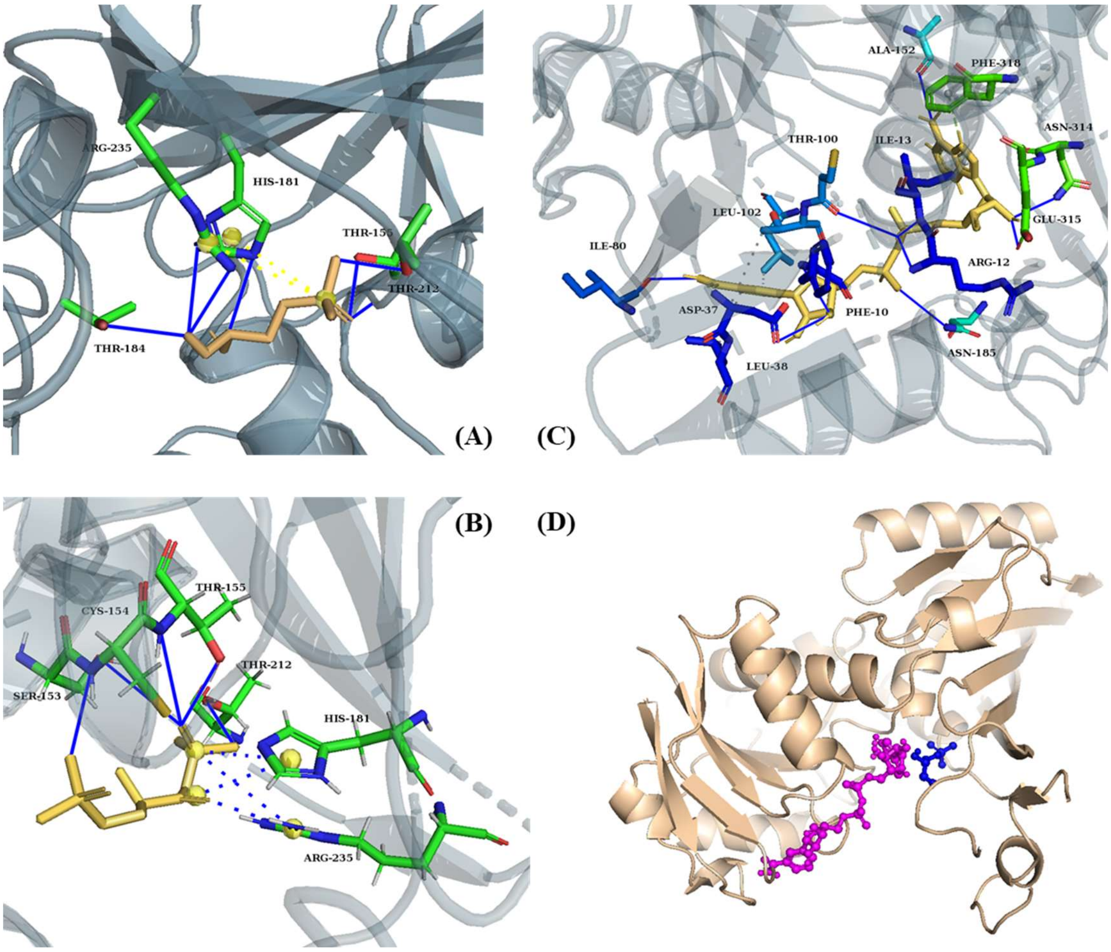

Figure S2. Molecular docking predictions for the Gap of *Pseudomonas plecoglossicida* JUIM01 with (A) glyceraldehyde-3-phosphate, (B) 1,3-bisphosphoglycerate, (C) NAD, and (D) both glyceraldehyde-3-phosphate (blue) and NAD (purple).

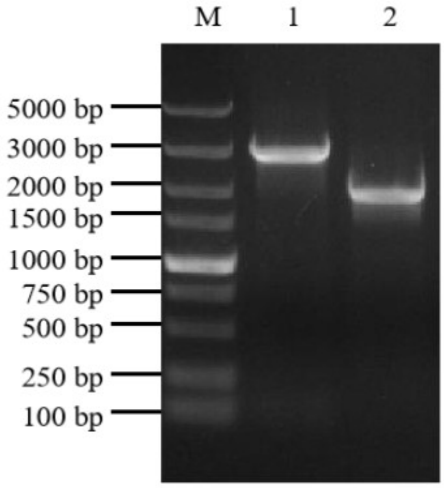

Figure S3. PCR validation of *gapA* knockout of *Pseudomonas plecoglossicida* JUIM01.

M, DL 5,000 DNA Marker; Lane 1, JUIM01; Lane 2, JUIM01Δ*gapA*.

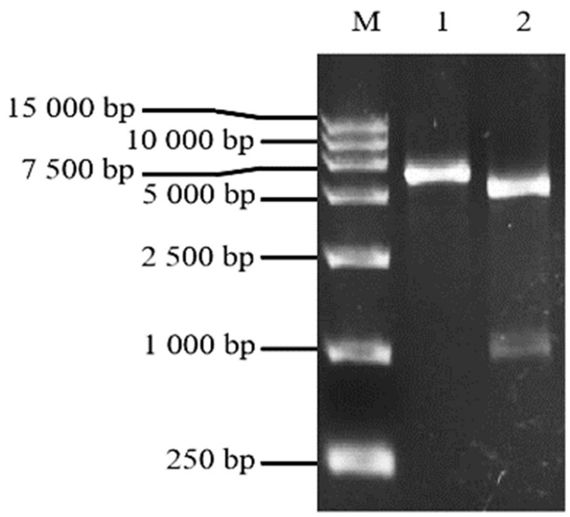

Figure S4. Restriction enzyme digestion verification of the recombinant expression plasmid pBBR1MCS-2-*gapA* for *gapA* complementation. The recombinant plasmid pBBR1MCS-2-*gapA* was verified through the digestion of the gene fragment with *Bam*H I and *Hin*d III. M, DL 15,000 DNA Marker; Lane 1, the single enzyme (*Bam*H I) digestion product of the recombinant plasmid; Lane 2, the double enzyme (*Bam*H I/*Hin*d III) digestion products of the recombinant plasmid.
